# Hospitalization of unintentional fall injuries in Kuwait: a national database study

**DOI:** 10.1186/s12889-021-11358-8

**Published:** 2021-07-10

**Authors:** Islam Kamal Ibrahim, Fatima AlAsoomi

**Affiliations:** National Center for Health Information, Ministry of Health, Kuwait City, Kuwait

**Keywords:** Falls, Hospitalization, Inpatient service days, Kuwait, Fractures, Utilization, Burden, Length of stay

## Abstract

**Background:**

Accidental falls are a major cause of morbidity placing pressure on hospital capacity and utilizing costly services. Evaluating the burden of falls is key for planning, implementation, and evaluation of prevention strategies. To date, no studies have been published on accidental falls at the population level in Kuwait. We studied the burden of accidental falls on public hospital inpatient capacity in Kuwait and identified the subgroups with the highest utilization of inpatient service days.

**Methods:**

From the national database of inpatient hospitalizations, we selected hospitalizations of patients admitted to Kuwait’s public hospitals for unintentional injury caused by an accidental fall from 1 January through 31 December 2016. We studied the number of inpatient service days (bed days), length of stay (LOS), and number of hospitalizations by age group, gender, and nationality. Mann–Whitney, Kruskal-Wallis, and Chi square tests were used for comparison. Logistic regression was used to quantify the risk of prolonged LOS and fractures among fall-related hospitalizations.

**Results:**

Accidental falls were responsible for 2.9% of inpatient hospitalizations, 3.7% of inpatient service days (61,140 days) with an ALOS of 9.1 days in Kuwait’s public hospitals in 2016. Accidental falls were responsible for 4.6% of older adult service days, and an even higher 5.6% of older women service days. In the age group 13–64, fall-related service days for non-Kuwaitis (5.7%) were more than triple those for Kuwaitis (1.8%) with a substantial percentage among male non-Kuwaitis (8.1%). The risk factors for exceeding the national ALOS for fall-related hospitalizations were female gender (OR 1.36), age 65 and older (OR 9.72), age 13–64 (OR 5.20), being non-Kuwaiti (OR 1.39), sustaining a femur fracture (OR 11.67), and undergoing surgery (OR 2.63). Fall-related hospitalizations associated with a higher risk of fractures were females (OR 1.22), patients 65 years and older (OR 5.09), patients aged 13–64 (OR 3.65), and non-Kuwaitis (OR 1.28).

**Conclusions:**

Accidental falls impose a considerable burden on inpatient service utilization in Kuwait. This varies by age, gender, and nationality. To reduce this burden, prevention programs should target working-age non-Kuwaiti males and older females.

## Background

Falls are a major cause of injury-related morbidity and mortality. Every year, around 172 million injuries globally are caused by falls, 37 million of which warrant medical care [1, 2]. A non-fatal fall can be a life-changing experience for an individual and their family causing disability, depression, considerable out-of-pocket health expenditure, and loss of income potentially leading to household impoverishment. The burden of falls extends to the health system as the resulting injuries place pressure on hospital capacity and utilize costly surgical and rehabilitative services. At a societal level, falls are associated with loss of productivity due to absenteeism, disability, and time needed to care for the injured person as well as the expenses of social care services [3–6].

The burden imposed by a fall does not only depend on the severity of the fall, but also on the frailty of the individual who falls. A young healthy construction worker who suffers a fall from height at work incurs a substantial cost, but so does an older woman who suffers a simple fall on the same level at home. The risk of a fall and subsequent injury is influenced by multiple factors; age, gender, comorbidities, medications, and occupation may all play a role [1, 7].

Evidence from clinical trials has shown that falls can be preventable in vulnerable populations such as older adults [8]. Evaluating the burden of falls is key for planning and implementation of fall prevention strategies. Data is needed to quantify the magnitude of the problem to make a case for investment in fall prevention strategies to policy makers; to prioritize those at risk to help allocate limited resources; and finally, to evaluate the effectiveness of the implemented interventions. From a health system perspective, monetary cost could be the most straightforward means for evaluating the burden of falls. However, this requires detailed cost data which is not always available. Morbidity measures, on the other hand, are more accessible as data on number of inpatient admissions, service days, and length of stay are routinely collected. These measures provide an alternative way for evaluating the utilization of services and burden on the system since they impact the operating cost of healthcare facilities [9].

As the world struggles with the COVID-19 pandemic, inpatient health services have been thinly stretched in most countries with insufficient bed capacity to accommodate the increasing number of cases. We therefore need to be more efficient in utilizing the capacity of inpatient service days. By reducing the proportion attributed to preventable conditions, we can make more service days available.

Kuwait is a high-income Middle-East country with a GDP per capita of around US$24,700 in 2016 [10]. In the same year, it had a population of 4.3 million of whom 30.5% were Kuwaiti [11]. The Ministry of Health is the primary funder and provider of healthcare in the country through its public hospitals [12]. Healthcare expenditure has soared over the past decades prompting governments to invest in evidence-based cost-effective programs to reduce the burden of potentially preventable hospitalizations. The total cost of healthcare services in Kuwait has increased dramatically from around 970 million Kuwaiti Dinars (around US$3.2 billion) in the fiscal year 2011/2012 (861US$ per person) to 1.7 billion KD (US$5.8 billion) in 2015/2016 (1277US$ per person) [13].

To date, no population-level studies have been published on accidental falls as a cause of injury in Kuwait. This research aimed to study the burden of accidental falls on inpatient capacity of Kuwait’s public hospitals through quantifying the fall-related hospitalizations, inpatient service days (bed days), and the length of stay attributed to accidental falls. Fall outcomes that are expected to impact hospital utilization namely fractures, need for surgery, and inpatient mortality were examined. We also aimed to identify the population subgroups with the highest utilization of inpatient service days.

## Methods

This is a nationally-representative retrospective database study using data available in the national database of inpatient hospitalizations maintained by the National Center for Health Information, Ministry of Health, Kuwait. The database contains a record for each inpatient hospitalization from all public hospitals in Kuwait. In 2016, 233,943 inpatients were discharged from Kuwait’s 17 public hospitals [11]. In this study, the term hospitalization was used to refer to hospital discharges from public hospitals in Kuwait.

Every record in the national database includes: patient demographic data; diagnosis codes for the principal diagnosis and one additional diagnosis; an ICD-10 code for the external cause of injury in cases of injury; surgical procedure codes for a maximum of two surgical procedures per patient; and discharge status. Unmodified WHO ICD-10 codes are used for coding diagnoses and external causes of injury while the International Classification of Procedures in Medicine (ICPM) codes are used for procedures.

**Fig. 1:**
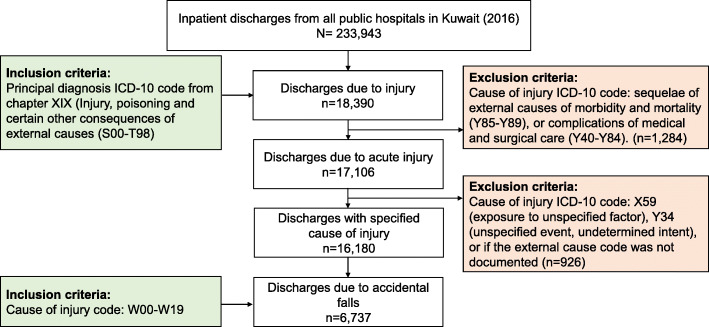
Flowchart for record selection of accidental fall-related hospitalizations from the national database of public hospital discharges

Figure 1 summarizes the record selection process. Records of hospitalizations in all public hospitals from 1 January through 31 December 2016 with a principal diagnosis of injury were obtained from the national database. The principal diagnosis in the Kuwait national database refers to the condition determined after study to be the reason for admission. These were identified by ICD-10 codes from chapter XIX (Injury, poisoning and certain other consequences of external causes: S00-T98). Acute injury cases were then selected based on cause of injury codes. Hospitalizations were excluded if the cause of injury code indicated: sequelae of external causes of morbidity and mortality (Y85-Y89), or complications of medical and surgical care (Y40-Y84). To identify acute injury cases with a documented cause of injury, hospitalizations were excluded if the cause of injury code was X59 (exposure to unspecified factor), Y34 (unspecified event, undetermined intent), or if the external cause code was not documented. To identify hospitalizations due to accidental falls, we selected all hospitalizations with the ICD-10 cause of injury codes W00-W19. These are hospitalizations of patients admitted to the hospital with the principal diagnosis of unintentional injury caused by an accidental fall; referred to throughout this paper as fall-related hospitalizations. A record with an ICPM code from 5010 through 5999 was considered to have undergone a surgical procedure.

Statistical analysis was performed using Statistical Package for the Social Sciences (IBM-SPSS version 27, Chicago, Il, USA) and MedCalc for Windows, version 19.4 (MedCalc Software, Ostend, Belgium). Length of stay was tested for normality using Kolmogorov–Smirnov test, was found to be skewed to the right, and was described using median (Mdn) and interquartile range (IQR). Mann–Whitney U test was used for comparing two groups and Kruskal-Wallis test for multiple groups with corrected post hoc pairwise comparisons. The mean and standard deviation (mean ± SD) were also reported for comparison with other studies and for ease of reporting to policy makers. Categorical data was described using frequencies and percentages and was analyzed using Chi-square test. Two logistic regression models were built to quantify the risk of prolonged LOS and the risk of fractures among fall-related hospitalizations while controlling for other risk factors. Based on the variables routinely collected in the national database, the risk factors available for quantifying the risk of prolonged LOS were gender, age, nationality, undergoing surgery, and sustaining a fracture. For the model studying the risk of sustaining a fracture, the risk factors were gender, age, and nationality. Prolonged LOS was defined in our study as that exceeding the national average length of stay (ALOS) for all public hospitals in Kuwait in 2016 (excluding psychiatry and rehabilitation hospitals) which was 6 days [11]. Relative risk (with 95% confidence interval) was calculated to quantify the risk of negative outcomes attributed to accidental falls relative to other causes. A *p* value <.05 was considered statistically significant.

## Results

A total of 233,943 inpatients were discharged from Kuwait’s public hospitals in 2016. Acute injury was the principal diagnosis in 17,106 hospitalizations (7.3%) of whom 16,180 (94.6%) had a specified external cause of injury. Accidental falls (*n* = 6737) were the most common cause of injury representing 41.6% of hospitalizations with a specified cause of injury; and 2.9% of the total 233,943 hospitalizations (Fig. 1).

Of fall-related hospitalizations, 68.2% were male, 64.6% were aged 13–64, and 62.8% were non-Kuwaiti. The median LOS for all fall hospitalizations was 4 (IQR 2–9) days with an average of 9.1 ± 47.7 days. Length of stay was significantly different between the three age groups, *p* < .001. Post-hoc pairwise comparison shows that older adults (65 years and older) had longer length of stay (Mdn 10 days; IQR 3,22) than those aged 13–64 (Mdn 5 days; IQR 2,9), who in turn stayed longer than those aged below 13 years (Mdn 2 days; IQR 1,3). Non-Kuwaitis also had a longer length of stay (Mdn 4 days; IQR 2,9) than Kuwaitis (Mdn 3 days; IQR 1,7), which was statistically significant, *p* < .001 (Table 1).

**Table 1:** Number of fall-related hospitalizations and their length of stay, Kuwait public hospitals, Jan – Dec 2016

	Fall-related hospitalizations	LOS in days		*p* value*
	n (%)	Median (IQR)	Mean (SD)	
Gender				
Male	4595 (68.2)	4 (2,8)	8.5 (56.2)	.005^†^
Female	2142 (31.8)	4 (2,11)	10.2 (19.3)	
Age group				
0–12	1624 (24.1)	2 (1,3)	3.5 (12.3)	<.001^‡^
13–64	4353 (64.6)	5 (2,9)	10.2 (58.2)	
65+	760 (11.3)	10 (3,22)	15.6 (19.0)	
Nationality				
Kuwaiti	2504 (37.2)	3 (1,7)	7.6 (16.1)	<.001^†^
Non-Kuwaiti	4233 (62.8)	4 (2,9)	9.9 (58.9)	
Total fall-related hospitalizations	6737 (100)	4 (2,9)	9.1 (47.7)	

* *p* value for comparing median LOS

^†^Mann–Whitney U test

^‡^Kruskal-Wallis test

Table 2 presents the percentages of fall-related inpatient service days in Kuwait’s public hospitals in 2016. For each population subgroup, the percentage is calculated out of the total number of service days due to all causes of admission for this subgroup. Accidental falls were responsible for 3.7% of all inpatient service days. For hospitalized male patients, falls accounted for 4.5% of service days compared to only 2.8% for females. However, stratifying the results by age, reveals that among adults 65 years and older, females had a higher percentage of fall-related service days (5.6%) compared to males (3.6%). Overall, older adults had the highest fall-related service days (4.6%), followed by the age group 13–64 (4.0%), and children below 13 (1.9%). In the age group 13–64, which includes those of working age, further stratification by nationality shows that the service days attributed to falls among non-Kuwaitis (5.7%) were more than triple those among Kuwaitis (1.8%) with falls accounting for a substantial percentage of service days among male non-Kuwaitis (8.1%).

**Table 2:** Inpatient service days for fall-related hospitalizations (frequency and percentage out of total service days for all causes of admission in each group)

	Malen (%)	Femalen (%)	Totaln (%)
Total hospitalizations for all causes of admission	877,442 (100)	769,608 (100)	1,647,050 (100)
Total fall-related hospitalizations	39,207 (4.5)	21,933 (2.8)	61,140 (3.7)
Fall-related hospitalizations per age group (years)^a^			
0–12	3437 (2.0)	2277 (1.6)	5714 (1.9)
13–64	31,455 (5.3)	12,149 (2.4)	43,604 (4.0)
*Kuwaiti*	*4,541 (1.8)*	*4,073 (1.9)*	*8,614 (1.8)*
*Non-Kuwaiti*	*26,914 (8.1)*	*8,076 (2.9)*	*34,990 (5.7)*
65+	4315 (3.6)	7507 (5.6)	11,822 (4.6)

^a^Percentage of fall-related inpatient service days for a subgroup = $\frac{inpatient\kern0.17em service\kern0.17em days\;\mathrm{attributed}\kern0.17em \mathrm{to}\kern0.17em \mathrm{falls}\;\mathrm{as}\;\mathrm{the}\kern0.17em \mathrm{cause}\kern0.17em \mathrm{of}\kern0.17em \mathrm{admission}\; in\;a\; subgroup}{total\kern0.17em inpatient\kern0.17em service\kern0.17em days\; due\; to\; all\; causes\kern0.17em of\kern0.17em admission\kern0.17em in\kern0.17em the\kern0.17em samesubgroup}\times 100$

In 2016, there were 146 in-hospital deaths among injury-related hospitalizations, 46 (31.5%) of them were fall-related. Relative to other causes of injury, falls were associated with a significantly higher risk of fractures (RR 2.20, 95% CI 2.13, 2.29), undergoing a surgical procedure (RR 1.43, 95% CI 1.37, 1.49), and prolonged LOS (RR 1.27, 95% CI 1.21, 1.34). On the other hand, falls had a significantly lower risk of inpatient death relative to other external causes which include road traffic accidents (RR 0.64, 95% CI 0.46, 0.91), (Table 3).

**Table 3:** Outcome of fall-related hospitalizations (*n* = 6737) relative to other causes of injury (*n* = 9443) among all injury-related hospitalizations (*n* = 16,180)

	Other external causes n (%)^a^	Falls n (%)^a^	RR (95% CI)	*p* value
Fracture				
No	6566 (74.8)	2212 (25.2)	1.0	
Yes	2877 (38.9)	4525 (61.1)	2.20 (2.13, 2.29)	<.001
Surgery				
No	6824 (62.7)	4068 (37.3)	1.0	
Yes	2619 (49.5)	2669 (50.5)	1.43 (1.37, 1.49)	<.001
Inpatient death				
No	9343 (58.3)	6691 (41.7)	1.0	
Yes	100 (68.5)	46 (31.5)	0.64 (0.46, 0.91)	.013
LOS				
≤ 6 days	7010 (60.8)	4526 (39.2)	1.0	
> 6 days	2433 (52.4)	2211 (47.6)	1.27 (1.21, 1.34)	<.001

^a^Percentages calculated from row totals

The significant independent risk factors for prolonged LOS among fall-related hospitalizations are presented in Table 4. The most important risk factors were sustaining a femur fracture (OR 11.67, 95% CI 9.18,14.84), and being 65 years and older (OR 9.72, 95% CI 7.58, 12.45 relative to children). The multiple logistic regression model also showed that female gender (OR 1.36, 95% CI 1.19, 1.55), age group (13–64) (OR 5.20, 95% CI 4.27, 6.34), sustaining a fracture other than femur fracture (OR 1.89, 95% CI 1.63, 2.19), undergoing surgery (OR 2.63, 95% CI 2.33, 2.97), and being non-Kuwaiti (OR 1.39, 95% CI 1.21, 1.59) were independently associated with a significantly higher odds of prolonged LOS.

**Table 4:** Risk factors for prolonged LOS (*n* = 2211) among fall-related hospitalizations (*n* = 6737)

	LOS > 6 days n (%)	Adjusted OR (95% CI)	*p* value
Gender			
Male	1419 (30.9)	1.0	
Female	792 (37.0)	1.36 (1.19, 1.55)	<.001
Age (years)			
0–12	149 (9.2)	1.0	
13–64	1618 (37.2)	5.20 (4.27, 6.34)	<.001
65+	444 (58.4)	9.72 (7.58, 12.45)	<.001
Nationality			
Kuwaiti	682 (27.2)	1.0	
Non-Kuwaiti	1529 (36.1)	1.39 (1.21, 1.59)	<.001
Fracture			
No	315 (14.2)	1.0	
Fracture of femur^a^	501 (75.9)	11.67 (9.18, 14.84)	<.001
Other fracture	1395 (36.1)	1.89 (1.63, 2.19)	<.001
Surgery			
No	942 (23.2)	1.0	
Yes	1269 (47.5)	2.63 (2.33, 2.97)	<.001

^a^Includes hip fracture

Among fall-related hospitalizations, female gender (OR 1.22, 95% CI 1.08, 1.37), older age (65 and older) (OR 5.09, 95% CI 4.15, 6.24), age group 13–64 (OR 3.65, 95% CI 3.21, 4.41), and being non-Kuwaiti (OR 1.28, 95% CI 1.14, 1.44) were all significant independent risk factors for sustaining a fracture (Table 5).

**Table 5:** Risk factors for sustaining a fracture (*n* = 4525) among fall-related hospitalizations (*n* = 6737)

	Fracture n (%)	Adjusted OR (95% CI)	*p* value
Gender			
Male	3063 (66.7)	1.0	
Female	1462 (68.3)	1.22 (1.08, 1.37)	.001
Age (years)			
0–12	693 (42.7)	1.0	
13–64	3227 (74.1)	3.65 (3.21, 4.41)	<.001
65+	605 (79.6)	5.09 (4.15, 6.24)	<.001
Nationality			
Kuwaiti	1514 (60.5)	1.0	
Non-Kuwaiti	3011 (71.1)	1.28 (1.14, 1.44)	<.001

A total of 4525 fall-related hospitalizations sustained fractures (67.2%) with an overall ALOS of 11.1 ± 51.1 days (Mdn 5, IQR 2,11). This was significantly longer than those without a fracture 4.9 ± 15.5 days (Mdn 2, IQR 1,4), *p* < .001. Of hospitalizations who sustained a fracture, 210 (5.9%) had two or more fractures. Table 6 shows that the most common fractures were those of the lower leg including ankle (23.3%), followed by forearm (19.9%), and femur (15.1%). The longest ALOS was that of femur fractures with 20.1 ± 22.3 days. The distribution of fracture sites differed by age group. Among patients 65 years and older, femur fractures (which include hip fractures) were the most common (48.3%), followed by lower leg (including ankle) (13.4%) and lumbar spine and pelvis (12.2%). In the 13–64 age group, the most common fractures were those of lower leg, including ankle (27.3%) followed by forearm (21.0%). In children below 13, fractures of shoulder and upper arm were the most common (31.5%) followed by forearm (26.7%).

**Table 6:** Fractures by body region among fall-related hospitalizations ^a^

Fracture site	0–12 years	13–64 years	65+ years	Total	LOS in days
	n (%)	n (%)	n (%)	n (%)	Mean (SD)
Lower leg, including ankle	93 (13.6)	846 (27.3)	78 (13.4)	1017 (23.3)	13.8 (116.7)
Forearm	183 (26.7)	651 (21.0)	38 (6.5)	872 (19.9)	5.5 (8.3)
Femur	104 (15.2)	274 (8.8)	282 (48.3)	660 (15.1)	20.1 (22.3)
Shoulder and upper arm	216 (31.5)	320 (10.3)	66 (11.3)	602 (13.8)	6.2 (9.9)
Foot, except ankle	12 (1.8)	352 (11.3)	12 (2.1)	376 (8.6)	9.5 (22.5)
Lumbar spine and pelvis	13 (1.9)	260 (8.4)	71 (12.2)	344 (7.9)	11.9 (19.4)
Skull and facial bones	47 (6.9)	184 (5.9)	2 (0.3)	233 (5.3)	4.0 (4.5)
Rib(s), sternum and thoracic spine	9 (1.3)	103 (3.3)	22 (3.8)	134 (3.1)	8.9 (14.8)
Neck	4 (0.6)	46 (1.5)	6 (1.0)	56 (1.3)	19.6 (40.4)
Spine, level unspecified	0 (0.0)	37 (1.2)	6 (1.0)	43 (1.0)	9.2 (13.5)
Wrist and hand level	3 (0.4)	29 (0.9)	1 (0.2)	33 (0.8)	3.9 (2.8)
Other region^b^	1 (0.1)	2 (0.1)	0 (0.0)	3 (0.0)	1.7 (1.2)
Total	685 (100)	3104 (100)	605 (100)	4373^c^ (100)	–

^a^The database allows only two diagnosis codes so in case of more than two fractures, ICD-10 code for “Fractures involving multiple body regions” is documented without specifying each fracture

^b^Includes fracture of lower limb, level unspecified, and fracture of upper limb, level unspecified

^c^For the 4525 hospitalizations with fractures, 210 had one ICD code for multiple fractures (excluded from this table), and 58 hospitalizations had two fracture diagnoses with an ICD-10 code for each fracture bringing the total number of fractures to 4373

## Discussion

This study showed that falls consumed 61,140 inpatient service days (3.7% of all service days) in public hospitals in Kuwait in 2016. Compared to other causes of injury, falls were associated with a higher risk of fractures, and undergoing surgery. It also showed that falls were the most common cause of injury-related hospitalizations (41.6%). This is consistent with evidence from the US where falls were also the most common mechanism of injury among traumatic injury hospitalizations (47.3%) [14].

Our findings show that twice as many males were admitted for a fall as females. Out of all causes of admission, falls accounted for a higher percentage of inpatient service days among males (4.5%) compared to females (2.8%). This is consistent with existing evidence that males are generally more likely to suffer injuries, including fall-related injuries, and their injuries tend to be more serious [14–16]. This difference in gender is more noticeable in working-age adults as males are more likely to take up physically demanding jobs with higher risk of injury. In this study, the age group 13–64 represented the highest proportion of fall-related hospitalizations (64.5%). This is consistent with results reported from Iran where those in the same age group represented 64.8% of all fall injuries admitted to the ED [17] as well as from the United Arab Emirates (UAE) where 68% of those admitted for a fall were aged 20–54 years [16].

Understanding population demographics in Kuwait is key to interpreting our results where age, gender, and nationality are concerned. Around 70% of Kuwait’s population are expatriates; 85% of whom are of working age (15–64 years). Males make up 70% of working age expatriates; many are manual laborers which may make them more prone to injury [11]. The distribution of the labor force in Kuwait in 2016 by economic activity shows that 72% of the expatriate labor force, compared to only 3% of the Kuwaiti labor force, work in “manufacturing, construction, wholesale and retail, repair of motor vehicles and motorcycles, other services, and the private household sector” [18]. This may explain why the majority of falls were non-Kuwaitis. It also may explain why fall-related service days for non-Kuwaitis in the age group 13–64 (5.7%) were more than triple those for Kuwaitis (1.8%). Further stratification by gender reveals an even higher percentage among male non-Kuwaitis (8.1%). These findings are consistent with findings from the UAE, which has a comparable demographic profile, where non-nationals represented 84.5% of patients admitted for a fall; 98.4% of whom fell at work [16].

Moreover, compared to Kuwaitis, non-Kuwaitis admitted for a fall were more likely to have sustained a fracture. They also had a significantly longer ALOS (10.2 days) since their falls are presumed to be work-related and therefore may be more serious such as fall from height among construction workers. This supports findings from another study on reported medico-legal cases of accidental deaths in Kuwait which reports that 78% of fall from height deaths were among those aged 20–59 years; 85% of whom were expatriates [19]. Further studies are needed to investigate the place of occurrence and the activity of the injured person at the time of fall to further examine these findings.

Life expectancy has dramatically improved throughout the world and Kuwait is no exception. Between 1960 and 2016, life expectancy at birth has steadily increased from 60.4 to 80.8 years [11, 20]. Aging is associated with multiple chronic conditions and comorbidities such as muscle weakness, visual impairment, impaired balance or mobility, depression, cognitive impairment, and osteoarthritis [21–23]. These factors not only increase the risk of falling, but also complicate the seriousness of the resulting injury and treatment. A low-impact fall during normal daily activities in older people with comorbidities can cause serious injuries requiring medical attention with much longer and more expensive hospital stay than a similar fall in younger people [5]. A study in Saudi Arabia reported that 36% of fall-related injuries in older adults required hospitalization for more than 24 h [24]. Observations in a study from ten European countries estimated that patients older than 65 years use up 46% of the cost of injury-related hospital admissions [25].

Our findings show that falls utilized a considerable share of the inpatient service days of older adult hospitalizations (65 years and older). Relative to younger age groups, older adults ranked least in terms of number of fall-related hospitalizations (11.3%) yet, they had the longest ALOS (15.6 days) as well the highest percentage of inpatient service days (4.6%) within their age group. Similar results were reported in Australia where fall-related injuries had an ALOS of 10 days and accounted for 4.2% of patient days for that group [26]. Comorbidities, including diabetes, play an important role in increasing the risk of hospitalization and prolonging LOS among older adult falls [27, 28]. The prevalence of diabetes in Kuwait’s adult population is 14.6% making it one of the top 20 countries in diabetes prevalence worldwide. This prevalence increases with age reaching 46.9% in those aged 60–69 [29, 30]. It was not possible to evaluate the relationship between diabetes and fall-related fractures or inpatient utilization in this study because data on comorbidities were not available. Research into the effect of comorbidities in older adult patients in Kuwait, including a special focus on diabetes, is needed to provide better understanding of factors affecting hospital utilization in this vulnerable group and to guide fall prevention measures.

Our findings show that 79.6% of patients 65 years and older hospitalized for a fall had sustained a fracture. This percentage was much higher than that reported by Australia (52%) but comparable to the UAE (81.9%) [26, 31]. Many factors contribute to the high risk of fractures among Kuwait’s older adults. Lack of physical activity and obesity are important risk factors for falls, fractures, and ensuing morbidity such as non-union of fractures and slower rehabilitation in this age group [32, 33]. Kuwait has the highest prevalence of physical inactivity in the world (67.0%) and ranks among the top ten countries in prevalence of obesity (40.3%) [34, 35].

In contrast to the younger age groups in our study, the gender difference was reversed in patients aged 65 years and older with a higher percentage of fall-related service days among females hospitalizations (5.6%) compared to males (3.6%) This indicates that falls may cause more serious injury in older women than in older men. This is consistent with the findings of a US study that fall-related diagnoses were generally higher among older women compared to men especially for fractures that were 2.2 times higher. The US study mentions differences in physical activity, and therefore muscle strength, as a possible explanation for the observed difference in older adults; with women being less physically active than men [36] In Kuwait, prevalence of obesity and low physical activity, which are already high in the population, further increase with age especially in women. Statistics in different age groups in both men and women in Kuwait show that women aged 60–69 have the highest percentage of low level of physical activity (88%), and obesity (71%) [29]. Furthermore, the combination of falls and osteoporosis, which is more prevalent in older women, increases their risk of fractures and complications [5].

The most common fractures among those 65 years and older in this study were femur fractures, which include hip fractures, (48%), followed by lower leg fractures (27%). The ALOS for femur fractures was 20 days, longer than any other fracture in this study. This is comparable to the 23 days reported for hip fractures in the UK [37]. This corroborates previous evidence from Europe in which hip fractures ranked first in terms of both incidence and cost per capita, followed by fractures of the knee/lower leg [25]. Another study in Senegal identified proximal femur fractures as the highest contributor to the cost of hospital treatment of fractures in older people [38]. Cost estimation of fall admissions in Kuwait is a topic that has not been addressed or published before. Presenting the cost of falls and the potential savings to policy makers could be a driving force for prevention programs.

Utilization of inpatient services was the least in children below 13. They represented 24.1% of fall hospitalizations and had the shortest ALOS of 3.5 days. In Iran, children below 13 represented 27.6% of falls admitted to the ED. [17] Since our study includes hospital inpatient admissions only, our numbers do not include less severe cases treated at the ED who did not require hospital admission. This may account for the lower proportion reported in our results compared to those in the aforementioned study. Falls consumed only 1.9% of service days in children below 13. They suffered significantly less fractures than older age groups with forearm fractures being the most common (27%), followed by those of lower leg (14%). A study from Sweden also reported distal forearm fractures as the most common fractures requiring admission among children below 19 seen for injuries at the ED (24%), followed by tibial/fibular shaft (13%) [39].

There are different approaches for evaluating the burden of falls. These include measures related to mortality, morbidity, composite (combining mortality and morbidity), and cost [9]. In the current study we used morbidity measures including inpatient hospitalizations, length of stay, and percentage of inpatient service days to evaluate the burden of falls on healthcare services as an indirect way of quantifying how much resources they use. We also identified serious outcomes that could increase resource utilization such as fractures and need for surgery. A longer LOS or a higher percentage of service days attributed to a certain medical condition reflects the complexity of its treatment and the higher utilization of resources. The decision to use the morbidity approach was based on data availability and quality as data on inpatient hospitalizations are routinely collected and readily available in the national database. Building capacities in health economics is needed to evaluate the burden of falls and other injuries in monetary terms.

This study has demonstrated that falls are a considerable public health problem and are associated with high utilization of public healthcare services in Kuwait. Our results show that falls in working-age non-Kuwaitis, especially males, and older people, especially females, utilize a considerably high proportion of inpatient services. Falls are preventable. Evidence from clinical trials shows that prevention programs for the population subgroups at high risk of falls, such as those 65 years and older, does not only decrease the risk of falls but also improves outcomes for those who have fallen [8]. Effective interventions are also available with respect to falls in the workplace. Prevention programs can improve workplace safety and reduce the risk of serious falls such as fall from height among construction workers [40–42].

The best return on investment in healthcare comes from prevention rather than treatment [43]. To reduce the burden on curative health services, policy makers need to focus on prevention rather than cure as that frees up capacity in health facilities and reduces future spending on disability and social care. Therefore, to reduce the burden of falls, fall prevention programs are needed and should target those imposing the biggest burden namely working-age non-Kuwaiti men, and older women. More studies are needed to identify mechanisms of injury and risk factors of falling in these two population subgroups particularly in the Kuwait context in order to tailor preventive programs to the more susceptible individuals.

### Strengths

This is the first study on accidental falls at the population level in Kuwait. Quantifying the burden of falls on public health services using morbidity-related measures in terms of their share of inpatient days and identifying the sectors of the population with the highest utilization of inpatient services are among the strengths. The existence of a national database of inpatient hospitalizations in addition to the presence of cause of injury ICD-10 codes has enabled us to use the database to study the whole population of fall-related hospitalizations making the results nationally-representative. Previous research on causes of injury in Kuwait was either limited to accidental deaths referred for medico-legal examination, or causes of construction site accidents [19, 44].

### Limitations

This study has some limitations. It focuses only on falls that required inpatient hospital admission. Indeed, some less severe falls may have been treated in other healthcare settings, such as the ED, but these are usually less-resource intensive than those requiring admission.

Our study did not include private and oil sector hospitals. However, in Kuwait, the public sector is the main provider of healthcare. In 2016, private hospitals were responsible for only 16% of orthopedic department discharges and 6.5% of ICU discharges [11].

Data on the specific number and location of fractures in cases of multiple fractures were not available because the national database of inpatient hospitalizations in Kuwait allows only one secondary diagnosis code per record. Adding fields for more diagnoses will provide data not only on individual injuries in cases of multiple injuries, but also on comorbidities and other medical conditions which could not be evaluated in this study. This is a missed opportunity as comorbid medical conditions that might have contributed to falls especially among older adult patients (65 years and older) such as diabetes were not available.

## Conclusions

Accidental falls in Kuwait are responsible for 2.9% of inpatient hospitalizations, 3.7% of inpatient service days (61,140 days) with an ALOS of 9.1 days. Compared to other causes of injury, fall-related hospitalizations have a higher risk of fractures, undergoing surgery, and exceeding the national ALOS. The risk factors for exceeding the national ALOS for fall-related hospitalizations are female gender, age 65 and older, age 13–64, being non-Kuwait, sustaining a fracture especially a femur fracture, and undergoing surgery. Fall-related hospitalizations associated with a higher risk of fractures were females, patients 65 years and older, patients aged 13–64, and non-Kuwaitis. In order to reduce the burden of falls on inpatient hospital utilization, fall prevention programs should target working-age non-Kuwaiti males and older females since falls consumed 8.1 and 5.6% of their inpatient service days respectively.

## Data Availability

The dataset used and analyzed for this study is available from the corresponding author Islam Ibrahim (e-mail islam.k.ibrahim@outlook.com) on reasonable request after obtaining required permits from the Standing Committee for Coordination of Health and Medical Research, MOH Kuwait.

## Abbreviations

ALOS: Average Length of Stay
CI: Confidence interval
ED: Emergency Department
GDP: Gross Domestic Product
ICD-10: International Statistical Classification of Diseases and Related Health Problems, tenth revision
ICPM: International Classification of Procedures in Medicine
ICU: Intensive Care Unit
IQR: Interquartile range
LOS: Length of Stay
Mdn: Median
OR: Odds Ratio
RR: Relative Risk
SD: Standard Deviation
UAE: United Arab Emirates
UK: United Kingdom

## References

[1] James SL, Lucchesi LR, Bisignano C, Castle CD, Dingels ZV, Fox JT, Hamilton EB, Henry NJ, Krohn KJ, Liu Z, McCracken D, Nixon MR, Roberts NLS, Sylte DO, Adsuar JC, Arora A, Briggs AM, Collado-Mateo D, Cooper C, Dandona L, Dandona R, Ellingsen CL, Fereshtehnejad SM, Gill TK, Haagsma JA, Hendrie D, Jürisson M, Kumar GA, Lopez AD, Miazgowski T, Miller TR, Mini GK, Mirrakhimov EM, Mohamadi E, Olivares PR, Rahim F, Riera LS, Villafaina S, Yano Y, Hay SI, Lim SS, Mokdad AH, Naghavi M, Murray CJL (2020). The global burden of falls: global, regional and national estimates of morbidity and mortality from the global burden of disease study 2017. Inj Prev.

[2] World Health Organization. Falls fact sheet. [Internet]. Geneva: World Health Organization; [updated 2018 Jan 16; cited 2020 Nov 10]. Available from: https://www.who.int/news-room/fact-sheets/detail/falls.

[3] Craig J, Murray A, Mitchell S, Clark S, Saunders L, Burleigh L (2013). The high cost to health and social care of managing falls in older adults living in the community in Scotland. Scott Med J.

[4] Florence CS, Bergen G, Atherly A, Burns E, Stevens J, Drake C (2018). Medical costs of fatal and nonfatal falls in older adults. J Am Geriatr Soc.

[5] World Health Organization (2007). WHO global report on falls prevention in older age.

[6] Hoffman GJ, Hays RD, Shapiro MF, Wallace SP, Ettner SL (2017). The costs of fall-related injuries among older adults: annual per-faller, service component, and patient out-of-pocket costs. Health Serv Res.

[7] Yeoh HT, Lockhart TE, Wu X (2013). Non-fatal occupational falls on the same level. Ergonomics..

[8] Guirguis-Blake JM, Michael YL, Perdue LA, Coppola EL, Beil TL (2018). Interventions to prevent falls in older adults: updated evidence report and systematic review for the US preventive services task force. JAMA..

[9] Hendrie D, Miller TR (2004). Assessing the burden of injuries: competing measures. Inj Control Saf Promot.

[10] Central Statistical Bureau (2017). Annual statistical abstract 2015-2016.

[11] National Center for Health Information (Health & Vital Statistics Division) (2016). Annual health report.

[12] Mossialos E, Cheatley J, Reka H, Alsabah A, Patel N (2018). Kuwait Health System Review.

[13] Ministry of Health (Budget control department) (2016). Cost analysis and performance evaluation for government health services 2015-2016.

[14] DiMaggio C, Ayoung-Chee P, Shinseki M, Wilson C, Marshall G, Lee D (2016). Traumatic injury in the United States: in-patient epidemiology 2000-2011. Injury..

[15] World Health Organization (2014). Injuries and violence: the facts 2014.

[16] Grivna M, Eid HO, Abu-Zidan FM (2014). Epidemiology, morbidity and mortality from fall-related injuries in the United Arab Emirates. Scand J Trauma Resusc Emerg Med.

[17] Rasouli MR, Saadat S, Haddadi M, Gooya MM, Afsari M, Rahimi-Movaghar V (2011). Epidemiology of injuries and poisonings in emergency departments in Iran. Public Health.

[18] Central Statistical Bureau (2017). Labor force survey 2016. [Internet].

[19] Al-Kandary N, Al-Waheeb S (2015). Patterns of accidental deaths in Kuwait: a retrospective descriptive study from 2003-2009. BMC Public Health.

[20] Kuwait Ministry of Health, World Health Organization (2013). World Health Survey in Kuwait.

[21] Tinetti ME, Kumar C (2010). The patient who falls: “It’s always a trade-off”. JAMA..

[22] Deandrea S, Lucenteforte E, Bravi F, Foschi R, La VC (2010). Risk factors for falls in community-dwelling older people: a systematic review and meta-analysis. Epidemiology..

[23] Gale CR, Cooper C, Aihie SA (2016). Prevalence and risk factors for falls in older men and women: the English longitudinal study of ageing. Age Ageing.

[24] Almegbel FY, Alotaibi IM, Alhusain FA, Masuadi EM, Al Sulami SL, Aloushan AF (2018). Period prevalence, risk factors and consequent injuries of falling among the Saudi elderly living in Riyadh, Saudi Arabia: a cross-sectional study. BMJ Open.

[25] Polinder S, Meerding WJ, van Baar ME, Toet H, Mulder S, Van Beeck EF, EUROCOST reference group (2005). Cost estimation of injury-related hospital admissions in 10 European countries. J Trauma.

[26] Australian Institute of Health and Welfare (2019). Trends in hospitalised injury due to falls in older people, 2007–08 to 2016–17. Injury research and statistics series no. 126.

[27] Yau RK, Strotmeyer ES, Resnick HE, Sellmeyer DE, Feingold KR, Cauley JA, Vittinghoff E, de Rekeneire N, Harris TB, Nevitt MC, Cummings SR, Shorr RI, Schwartz AV (2013). Diabetes and risk of hospitalized fall injury among older adults. Diabetes Care.

[28] Comino EJ, Harris MF, Islam MDF, Tran DT, Jalaludin B, Jorm L (2015). Impact of diabetes on hospital admission and length of stay among a general population aged 45 year or more: a record linkage study. BMC Health Serv Res.

[29] Kuwait Ministry of Health (2015). Eastern Mediterranean approach for control of non communicable diseases. Survey of risk factors for chronic non communicable diseases.

[30] Alkandari A, Longenecker JC, Barengo NC, Alkhatib A, Weiderpass E, Al-Wotayan R (2018). The prevalence of pre-diabetes and diabetes in the Kuwaiti adult population in 2014. Diabetes Res Clin Pract.

[31] Hefny AF, Abbas AK, Abu-Zidan FM (2016). Geriatric fall-related injuries. Afr Health Sci.

[32] Song S, MacDermid J, Grewal R (2013). Risk factors for falls and fragility fractures in community-dwelling seniors: a one-year prospective study. ISRN Rehabil..

[33] Prieto-Alhambra D, Premaor MO, Fina Avilés F, Hermosilla E, Martinez-Laguna D, Carbonell-Abella C, Nogués X, Compston JE, Díez-Pérez A (2012). The association between fracture and obesity is site-dependent: a population-based study in postmenopausal women. J Bone Miner Res.

[34] Guthold R, Stevens GA, Riley LM, Bull FC (2018). Worldwide trends in insufficient physical activity from 2001 to 2016: a pooled analysis of 358 population-based surveys with 1·9 million participants. Lancet Glob Health.

[35] Weiderpass E, Botteri E, Longenecker JC, Alkandari A, Al-Wotayan R, Al Duwairi Q (2019). The prevalence of overweight and obesity in an adult Kuwaiti population in 2014. Front Endocrinol.

[36] Stevens JA, Sogolow ED (2005). Gender differences for non-fatal unintentional fall related injuries among older adults. Inj Prev.

[37] Lawrence TM, White CT, Wenn R, Moran CG (2005). The current hospital costs of treating hip fractures. Injury..

[38] Diémé CB (2014). Economic cost of the treatment of fractures among old people: a preliminary study in Dakar teaching hospital. Geriatr Orthop Surg Rehabil.

[39] Hedström EM, Svensson O, Bergström U, Michno P (2010). Epidemiology of fractures in children and adolescents. Increased incidence over the past decade: a population-based study from northern Sweden. Acta Orthop.

[40] Evanoff B, Dale AM, Zeringue A, Fuchs M, Gaal J, Lipscomb HJ, Kaskutas V (2016). Results of a fall prevention educational intervention for residential construction. Saf Sci.

[41] Kaskutas V, Marie Dale A, Lipscomb H, Gaal J, Fuchs M, Evanoff B (2010). Fall prevention in apprentice carpenters. Scand J Work Environ Health.

[42] Goh YM, Goh WM (2016). Investigating the effectiveness of fall prevention plan and success factors for program-based safety interventions. Saf Sci.

[43] Mcdaid D, Kluge H, Figueras J (2018). Using economic evidence to help make the case for investing in health promotion and disease prevention.

[44] Al-Tabtabai HM (2002). Analyzing construction site accidents in Kuwait. Kuwait J Sci Eng.

